# An effective antimicrobial strategy of colistin combined with the Chinese herbal medicine shikonin against colistin-resistant *Escherichia coli*

**DOI:** 10.1128/spectrum.01459-23

**Published:** 2023-10-06

**Authors:** Yan Liu, Yue Wang, Jingchun Kong, Xianguo Jiang, Yijia Han, Luozhu Feng, Yao Sun, Lijiang Chen, Tieli Zhou

**Affiliations:** 1 Department of Clinical Laboratory, The First Affiliated Hospital of Wenzhou Medical University, and Key Laboratory of Clinical Laboratory Diagnosis and Translational Research of Zhejiang Province, Wenzhou, Zhejiang, China; 2 Department of Medical Lab Science, School of Laboratory Medicine and Life Science, Wenzhou Medical University, Wenzhou, Zhejiang, China; JMI Laboratories, North Liberty, Iowa, USA

**Keywords:** *Escherichia coli*, antibiotic resistance, biofilm, *mcr-1*, colistin-resistant, shikonin, synergistic effect

## Abstract

The treatment of bacterial infections has become more challenging due to the increasing prevalence of colistin-resistant (Col-R) pathogens. Shikonin is a Chinese herbal medicine that has been proven to have antibacterial properties. In recent years, many studies have shown that the combination of traditional antibiotics and non-antibacterial drugs can effectively treat infections caused by multidrug-resistant bacteria. This study evaluated the potential of shikonin in combination with colistin to inhibit colistin-resistant *Escherichia coli* (Col-R *E. coli*), and explored potential interaction. In checkerboard analysis, shikonin could significantly reduce the minimum inhibitory concentration (MIC) of colistin by two to eight times, enhancing the susceptibility of Col-R *E. coli* to colistin. Shikonin also increased the bactericidal effect of colistin against Col-R *E. coli* in 24 h time-kill assays, resulting in a >3 log-fold decrease in colony-forming units. *In vivo* tests showed that the combination of colistin and shikonin could significantly improve the survival rate of *Galleria mellonella*. In addition, confocal laser scanning microscopy and quantitative real-time polymerase chain reaction showed that colistin/shikonin could inhibit the formation of biofilms, and shikonin could down-regulate the transcription level of biofilm-regulated genes (*csgA*, *csgD*, *flhC*, *flhD*, *fliC*, *fliM*, *lsrK*, and *lsrR*). The synergistic mechanism of colistin and shikonin was further revealed by detecting membrane integrity, reactive oxygen species production, and *mcr-1* gene expression. Our findings indicate that the colistin/shikonin combination may be a promising alternative approach to treat Col-R *E. coli* infections.

**IMPORTANCE:**

Infections caused by multidrug-resistant *Escherichia coli* (MDR *E. coli*) have become a major global healthcare problem due to the lack of effective antibiotics today. The emergence of colistin-resistant *E. coli* strains makes the situation even worse. Therefore, new antimicrobial strategies are urgently needed to combat colistin-resistant *E. coli*. Combining traditional antibiotics with non-antibacterial drugs has proved to be an effective approach of combating MDR bacteria. This study investigated the combination of colistin and shikonin, a Chinese herbal medicine, against colistin-resistant *E. coli*. This combination showed good synergistic antibacterial both *in vivo* and *in vitro* experiments. Under the background of daily increasing colistin resistance in *E. coli*, this research points to an effective antimicrobial strategy of using colistin and shikonin in combination against colistin-resistant *E. coli*.

## INTRODUCTION

*Escherichia coli* (*E. coli*) is a Gram-negative bacterium (GNB) that commonly causes a variety of diseases in humans, including enteric/diarrhogenic diseases or extraintestinal infections (1). In recent years, *E. coli* has shown increasing prevalence worldwide and resistance to various antimicrobial agents, posing a serious threat to public health due to lack of effective treatment options (2, 3). More seriously, infections caused by *E. coli* are challenging to eradicate because of the formation of biofilms (4, 5). Biofilm-associated and multidrug-resistant (MDR) *E. coli* infections represent a major challenge in medical field (6).

Polymyxin is a class of cationic antimicrobial peptides, mainly including polymyxin B and polymyxin E (colistin). The development of antibiotic resistance has prompted reconsideration of polymyxin as the treatment of last resort against MDR GNB infections (7). Unfortunately, the increased and unreasonable use of polymyxin has led to the emergence of strains resistant to colistin (7, 8). Particularly colistin-resistant (Col-R) *E. coli*, its resistance and global spread pose a huge threat to global public health (9). The rise of Col-R *E. coli*, combined with its ability to cause various diseases and form resilient biofilms, represents an urgent problem needing new treatment solutions. Colistin remains critical as a last defense, so strategies to boost its effects or overcome resistance are urgently needed.

Shikonin is a compound with a naphthoquinone structure, which is the main active ingredient of a traditional Chinese herbal medicine made from the dried root of Lithospermum erythrorhizon (10). Shikonin has various pharmacological activities, including anti-inflammatory, anti-tumor, wound healing, and antimicrobial properties (11 – 13). Besides, many studies have revealed the potential of shikonin as an effective natural antibiotic. It has been proved that shikonin has outstanding antibacterial activity against *Staphylococcus aureus* (*S. aureus*), related to its ability to disrupt bacterial membrane integrity (14 – 16). And synergistic effects of shikonin in combination with conventional antibiotics against methicillin-resistant *Staphylococcus aureus* (MRSA) have also been demonstrated (15).

Given shikonin’s antimicrobial properties and ability to synergize with antibiotics, this study aimed to investigate its combination with colistin against colistin-resistant *E. coli* as well as the underlying cooperative mechanisms of the colistin/shikonin combination. This combination may help overcome both colistin-resistance and biofilm formation. Our study may provide an effective therapeutic idea for treating infections caused by Col-R *E. coli*.

## MATERIALS AND METHODS

### Strains and growth conditions

A total of eight clinical isolates of cCol-R *E. coli* were obtained from the First Affiliated Hospital of Wenzhou Medical University for use in this study. The *E. coli* ATCC 25922 was used as the quality control reference strain, which was purchased from the National Center of the Clinical Laboratory. All strains were stored in Luria-Bertani (LB) broth medium (Sigma-Aldrich, St. Louis, MO, USA) containing 30% glycerol at −80°C.

### Antimicrobial agents and reagents

Shikonin (≥98% purity) was purchased from MedChem Express Co., Ltd. (NJ, USA) and dissolved in 1% (vol/vol) dimethyl sulfoxide (Sigma-Aldrich). The antimicrobial agents used in this study, including cefotaxime, ceftriaxone, ciprofloxacin, levofloxacin, imipenem, gentamicin, amikacin, nitrofurantoin, trimethoprim, aztreonam, and colistin, were purchased from Wenzhou Kangtai Biological Technology Co., Ltd. (Zhejiang, China) and Solar Science & Technology Co., Ltd. Stock solutions and diluents of antibiotics were prepared according to the latest Clinical and Laboratory Standards Institute guidelines (CLSI 2022).

### Antimicrobial susceptibility testing

The minimum inhibitory concentrations (MICs) of commonly used antibiotics and shikonin against eight Col-R *E. coli* isolates were determined by broth microdilution method in cation-adjusted Mueller-Hinton Broth (CAMHB; Sigma-Aldrich) following the guidelines of CLSI 2022. After 18–20 h of incubation at 37°C, the MIC was defined as the minimum concentration of the drug that inhibited the visible growth of bacteria.

### Checkerboard assays

A checkerboard assay was performed to determine the interaction of colistin and shikonin according to Yu et al. (17). In checkerboard assays, colistin and shikonin were diluted twofold in 96-well plates with CAMHB to form a series of concentration gradients. The vertical wells of the 96-well plates were added with 50 µL shikonin (final concentration: 2–128 μg/mL), and then the horizontal wells were added with 50 µL colistin (final concentration: 0.125–128 μg/mL). Subsequently, a total of 100 µL of the bacterial suspensions (1.5 × 10^6^ CFU/mL) was added to 96-well plates containing colistin and shikonin. Finally, the plates were incubated at 37℃ for 18–20 h to determine the MIC values and to calculate the fractional inhibitory concentration index (FICI) (17). FICI was calculated as follows: FICI=MIC_combination-colistin_/MIC_alone-colistin_+MIC_combination-shikonin_/MIC_alone-shikonin_. The combination is considered synergistic when the FICI is ≤0.5, and additive when 0.5 < FICI ≤ 1. The experiments were performed in triplicate.

### Time-kill assays

The time-kill assay was performed to further determine the synergistic effect of colistin combined with shikonin observed in the checkerboard, as described with some modifications (18, 19). In short, the bacterial suspensions of 0.5 McFarland were firstly prepared from the 4 Col-R *E. coli* strains and inoculated into 10 mL LB broth (100 µL) containing either no drug (control), colistin (1 or 2 µg/mL), shikonin (32 µg/mL), or colistin/shikonin (combination treatment), respectively. The cultures were incubated at 37℃ with shaking at 200 rpm. Then, aliquots were taken out at 0, 4, 8, 12, and 24 h, serially diluted in sterile saline and coated on LB agar plates to count colony-forming units (CFUs) after overnight incubation at 37°C.

The bactericidal activity was defined as a ≥3 log10 CFU/mL reduction in 24 h, and the synergistic activity was defined as a ≥2 log10 reduction. The mean results were presented on a logarithmic scale with a standard deviation for each data point. The experiments were performed in triplicate.

### Confocal laser microscopy for visualization of biofilms

Next, we examined biofilm formation in DC 4887 using confocal laser scanning microscopy (CLSM) (20). The concentrations of colistin and shikonin used in this study were determined based on the results of the checkerboard assay. In a nutshell, biofilms were grown on the coverslips placed at the bottom six-well plates containing colistin (2 µg/mL), shikonin (32 µg/mL) alone, or their combination at 37°C, biofilms grown in the absence of drugs served as control. After 24 h, the biofilms were washed two times with phosphate-buffered saline (PBS) to remove non-adherent cells. Biofilms were stained using the BacLight Live/Dead viability kit (L7012, Invitrogen, Thermo Fisher Scientific, Eμgene, OR, USA) according to the manufacturer’s instructions. Afterward, excess staining was removed by washing two times with PBS, and then the biofilms were imaged by CLSM (Nikon A1R, Japan).

### Quantitative real-time polymerase chain reaction for detecting the transcription levels of *E. coli* biofilm-related genes

According to the method described by Bai et al. (5), the effect of shikonin on the transcription of regulatory genes in *E. coli* DC4887 biofilm was investigated using quantitative real-time polymerase chain reaction (qRT-PCR). Bacterial suspensions were incubated with or without shikonin (32 µg/mL) at 37°C for 18 h. After 18 h, RNA was extracted using RNAiso Plus (TAKARA) for reverse transcription into cDNA using the PrimeScript RT reagent Kit (TAKARA). qRT-PCR was then conducted following the manufacturer’s instructions (SYBR Green kit, Applied Biosystems, USA). The 2^−∆∆Ct^ method was used to assess relative changes in gene transcription levels. The *gapA* gene was used as an internal control. The primers used in this study are listed in Table S1.

### *In vivo* infection model of *Galleria mellonella*

Then a *Galleria mellonella* (*G. mellonella*) infection model was established to further identify the synergistic effect of the combination *in vivo* (21). The larvae of four experimental groups (PBS group, colistin or shikonin monotherapy group, and colistin/ shikonin combined group) were infected with 10 µL bacterial suspension (1 × 10^7^ CFU/mL). Two hours after infection, 10 µL PBS or colistin or shikonin or the combination was administered, and the larvae injected with only PBS but no bacterial suspensions were used as a control group. DC 4887 was selected as the experimental strain. Larvae weighing between 250 and 350 mg were selected. Each group contained 10 larvae.

Larval survival was monitored for 5 days. Survival data were plotted using the Kaplan–Meier method and comparisons were made between groups using the log-rank test.

### *In vivo* evaluation of toxicity of shikonin on *G. mellonella*

Next, we evaluated drug toxicity using a method adapted from a previous study, with slight modifications (22). Briefly, 10 µL of shikonin at different concentrations (16–256 μg/mL) was injected through the last left pro-leg of larvae. Larvae injected with only 10 µL PBS served as control. Each group contained 10 larvae. Survival data analysis and comparison were made as above described.

### Outer membrane permeability assay

To investigate the interaction between colistin and shikonin, we analyzed membrane integrity. Outer membrane (OM) permeability was assessed using the fluorescence probe 1-N-phenylnaphthylamine (NPN) with minor modifications (23, 24). Single bacterial colony of each strain was selected for overnight culture in LB broth. Bacteria were then harvested by centrifugation at 3,000 × *g* for 10 min. After washing two times with PBS, bacteria were resuspended in PBS to an OD_600_ of 0.4. The bacterial suspension of two strains were then incubated with colistin (1 or 2 µg/mL), shikonin (32 µg/mL), or a combination of both. Bacterial suspensions incubated without any treatment worked as control. After 2 h, bacteria were harvested by centrifugation at 3,000 × *g* for 10 min. Cell pellets were washed and resuspended in PBS before the addition of NPN solution (final concentration, 30 µM). After co-incubation for 30 min at 37℃ in the dark, the fluorescence of NPN was monitored (λexc/λem: 350/420 nM) using a microplate reader (BioTek, Synergy, USA) to determine OM permeability.

### Inner membrane permeability assay

Inner membrane permeability was assessed to examine membrane integrity using Propidium Iodide (PI) (25). Bacterial suspensions (OD_600_ = 0.4) were treated and stained as described above, except that NPN was replaced with PI (final concentration, 50 μg/mL). Fluorescence of PI was visualized using an inverted fluorescence microscope (λexc/λem: 535/615 nM). Bacteria with damaged cell membranes stained with PI will show red fluorescence, whereas intact bacterial cells remain unstained (25)

### Intracellular reactive oxygen species detection

To detect the effect of colistin/shikonin on bacterial reactive oxygen species (ROS) production, we measured the ROS level using a fluorescent probe DCFH-DA, according to the previously reported protocol with some modifications (26). The ROS Assay Kit was purchased from Beyotime Biotechnology. Briefly, *E. coli* suspensions (OD_600_ = 0.3) were co-incubated with DCFH-DA probes (final concentration,10μM) for 45 min at 37°C in the dark to allow the probes to load into the cells. Cells were then washed two times with PBS to remove excess probes. Next, bacterial cells loaded with probes were resuspended in 1 mL PBS and treated with either single drugs (colistin or shikonin), colistin/shikonin combination, a positive control reagent (Rosup), or a PBS negative control and cells were incubated for 2 h. Finally, after 2 h of incubation, the fluorescence intensity was measured (λexc/λem: 488/535 nM) with a microplate reader (BioTek, Synergy, USA).

### qRT-PCR for detecting transcription levels of *mcr-1* gene

To assess the effect of shikonin on expression of the colistin resistance gene *mcr-1* in *E. coli*, transcription of *mcr-1* was analyzed by qRT-PCR using *mcr-1-*specific primers. Following a previously described method with minor modifications, the bacterial samples were processed (27). In brief, bacteria were cultured overnight in LB broth to early stage of logarithmic growth. Colistin, shikonin, a combination of both or no treatment (control) were then added to the corresponding concentration. After 6 h incubation at 37°C with shaking, RNA extraction and qRT-PCR reaction were performed as described above. Finally, expression of *mcr-1* was normalized to that of the *16S RNA* gene. The 2^−ΔΔCt^ method was used to identify statistically significant differences in gene expression. The primers used are listed in Table S1.

### Statistical analysis

All experiments were performed at least three times, and the data are presented as the mean ± S.D. The data were analyzed using a two-sample *t* test and log-rank test, and the differences among groups were evaluated using Dunnett’s multiple comparisons test. *P* < 0.05 was considered statistically significant; *, *P*＜ 0.05, **, *P*＜ 0.01, and ***, *P*＜ 0.001 for all analyses. GraphPad Prism 8.0 statistical software was used for statistical analyses.

## RESULTS

### MICs of commonly used antibiotics and shikonin against eight Col-R *E. coli* isolates

Table 1 shows the MIC values of antibiotics and shikonin against eight clinical isolates of *E. coli*. The results showed that these eight strains all exerted MDR phenotypes with varying degrees of decreased sensitivity to multiple antibiotics. These strains were all resistant to colistin with MIC_S_ ranging from 4 to 16 µg/mL, and MICs of shikonin against the eight strains were all greater than 256 µg/mL. In addition, these Col-R *E. coli* strains tested have been proven to harbor *mcr-1* in a previous study using PCR (28). The clinical background of eight Col-R *E. coli* isolates is shown in Table S2.

**TABLE 1:** Antibiotics and shikonin MICs of eight Col-R *E. coli* isolates^*a*^

Isolates	SKN MICs (μg/mL)	Antibiotic MICs (μg/mL)											*mcr-1*
		CTX	CRO	CIP	LVX	IPM	GEN	AMK	NIT	TMP	ATM	COL	
DC3539	>256	>32^R^	64^R^	>16^R^	16^R^	0.5^S^	>64^R^	32^I^	64^I^	>128^R^	64^R^	8^R^	＋
DC3599	>256	>32^R^	>64^R^	>16^R^	>16^R^	0.25^S^	32^R^	16^S^	64^I^	>128^R^	64^R^	4^R^	＋
DC3737	>256	>32^R^	>64^R^	>16^R^	>16^R^	64^R^	>64^R^	8^S^	128^R^	>128^R^	>64^R^	8^R^	＋
DC5286	>256	>32^R^	>64^R^	>16^R^	>16^R^	0.5^S^	4^S^	4^S^	16^S^	>128^R^	>64^R^	8^R^	＋
DC3846	>256	>32^R^	>64^R^	>16^R^	>16^R^	0.5^S^	>64^R^	4^S^	128^R^	>128^R^	>64^R^	8^R^	＋
DC4887	>256	>32^R^	>64^R^	8^R^	8^R^	0.5^S^	64^R^	4^S^	16^S^	>128^R^	1^S^	16^R^	＋
DC7333	>256	>32^R^	>64^R^	>16^R^	16^R^	16^R^	>64^R^	16^S^	128^R^	>128^R^	>64^R^	4^R^	＋
DC8277	>256	>32^R^	>64^R^	8^R^	8^R^	0.25^S^	16^R^	4^S^	16^S^	>128^R^	4^S^	8^R^	＋

^*a*^ CTX, cefotaxime; CRO, ceftriaxone; CIP, ciprofloxacin; LVX, levofloxacin; IPM, imipenem; GEN, gentamicin; AMK, amikacin; NIT, nitrofurantoin; TMP, trimethoprim; ATM, aztreonam; COL, colistin; SKN, shikonin; S-R represents the susceptible (S) breakpoint and resistant (R) breakpoint, according to CLSI supplement M100 (32nd edition) and EUCAST.

### Synergistic effects of shikonin in combination with colistin against eight *E. coli* strains

The synergistic antibacterial efficacy of colistin in combination with shikonin against Col-R strains was assessed through checkerboard assays (Table 2). As presented in Table 2, the colistin/shikonin combination displayed synergistic activity with FICI < 0.5 in six strains, and additive activity with 0.5 < FICI < 1 in DC 3599 and DC 5286. The MIC_S_ of colistin combined with shikonin (16 or 32 µg/mL) were four to eight times lower than that of colistin alone. In conclusion, the results of checkerboard experiment showed that colistin combined with shikonin had synergistic antibacterial effects on Col-R *E. coli*.The combination is considered synergistic when the FICI is ≤0.5, and additive when 0.5 < FICI ≤ 1. The experiments were performed in triplicate (17).

**TABLE 2:** Synergistic effect of colistin and shikonin on eight Col-R *E. coli* isolates^*a*^

Isolates	Colistin (μg/mL)		Shikonin (μg/mL)		FICI value	Interpretation
	MIC_alone_	MIC_combination_	MIC_alone_	MIC_combination_		
DC3539	8	1	>256	32	0.250	Synergistic
DC3599	4	2	>256	16	0.563	Additive
DC3737	8	2	>256	32	0.375	Synergistic
DC5286	8	4	>256	32	0.625	Additive
DC3846	8	2	>256	32	0.375	Synergistic
DC4887	16	2	>256	32	0.250	Synergistic
DC7333	4	1	>256	32	0.375	Synergistic
DC8277	8	2	>256	32	0.375	Synergistic

^*a*^ MIC, minimum inhibitory concentration; FICI, fractional inhibitory concentration index.

### Time-kill curves

**Fig 1:**
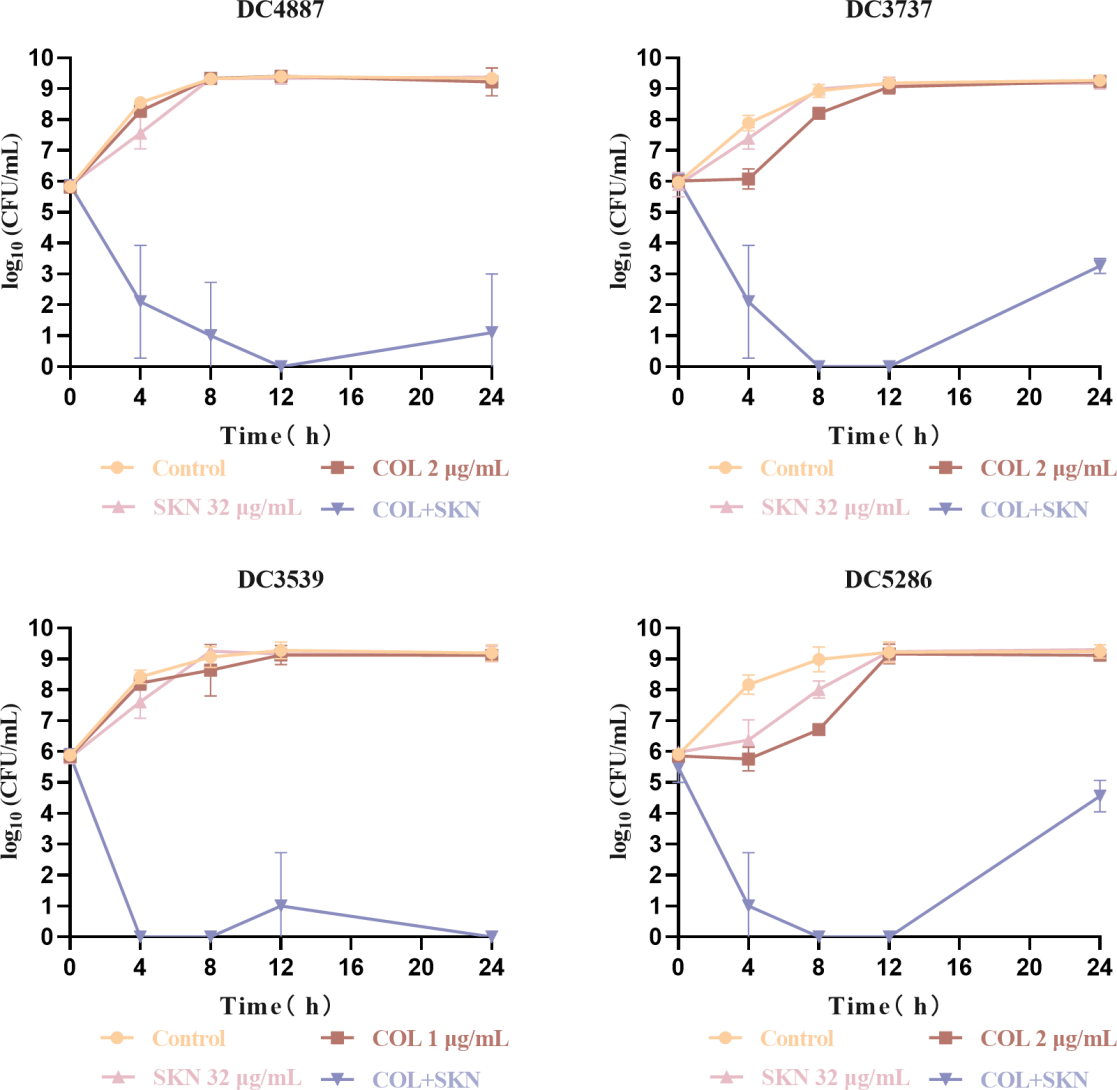
Time-kill curves of Col-R *E. coli* with colistin or shikonin monotherapy or combination treatment. Time-kill curves were obtained by counting-colony forming unit (CFU) at predetermined time points. Concentration of drugs used for the experiments: DC 4887, DC 3737, DC 5286 (COL: 2 µg/mL, SKN: 32 µg/mL), and DC 3539 (COL: 1 µg/mL, SKN: 32 µg/mL). Where the bactericidal activity was defined as a ≥3 log_10_ CFU/mL reduction in 24 h, and the synergistic activity was defined as a ≥2 log_10_ reduction (19). COL, colistin; SKN, shikonin.

To further validate the synergistic interaction of the colistin/shikonin combination observed in the checkerboard assays, time-kill assays were conducted against four Col-R strains (Fig. 1). The concentration of drug used for the time-kill curves was derived from the checkerboard analyses. The result is shown in Fig. 1. Bacterial cultures treated with either colistin or shikonin alone exhibited no bactericidal activity over 24 h. In contrast, co-treatment with colistin and shikonin combination at the same concentration as the monotherapy treatment resulted in a substantial reduction in CFUs (>3 log-fold) within 24 h. In summary, the combination elicited bactericidal activity against Col-R *E. coli* within 24 h at their respective non-inhibitory concentrations, providing further evidence for the synergistic effects of these two agents.

### The combination of shikonin and colistin impeded biofilm formation in Col-R *E. coli*

**Fig 2:**
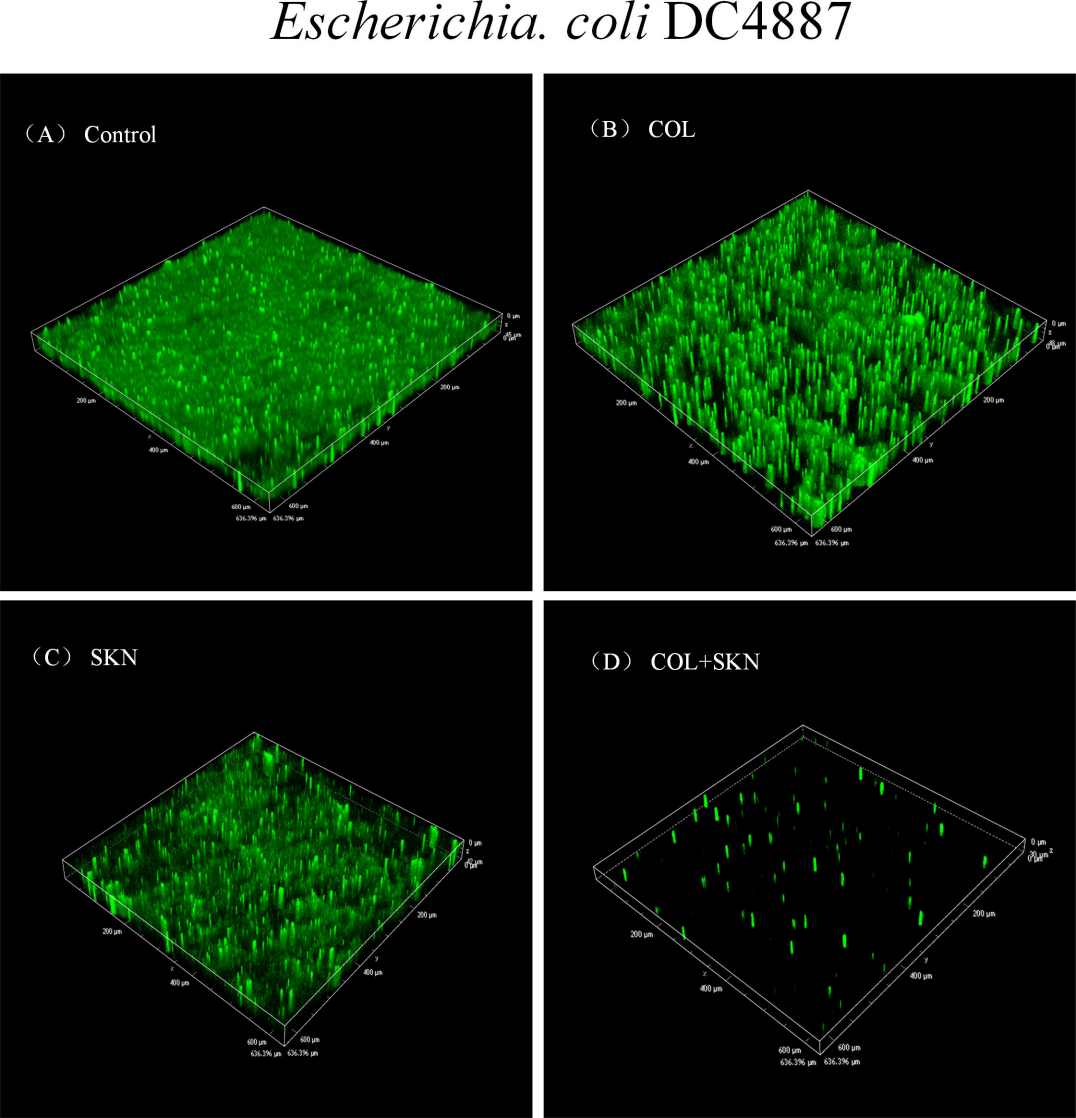
Effects of colistin/shikonin combination on biofilm formation measured by confocal laser scanning microscopy (CLSM) against DC 4887. (**A**) Cells without any treatments. (**B**) Cells treated with colistin (COL, 2 µg/mL). (**C**) Cells treated with shikonin (SKN, 32 µg/mL). (**D**) Cells treated with colistin/shikonin combination (COL +SKN).

We subsequently investigated the effects of colistin and shikonin alone or in combination on biofilm formation. Biofilms of DC 4887 were observed through CLSM to visualize the penetration of colistin/shikonin in parallel with live cells. As illustrated in Fig. 2, treatment with either colistin or shikonin alone only modestly inhibited the biofilms of strain DC4887 (Fig. 2B and C), while colistin/shikonin combination elicited a substantial diminution in biofilm formation (Fig. 2D) compared to the control or colistin or, shikonin monotherapy (Fig. 2A through C).

### Effects of shikonin on the transcription of biofilm-regulated genes in Col-R *E. coli*

**Fig 3:**
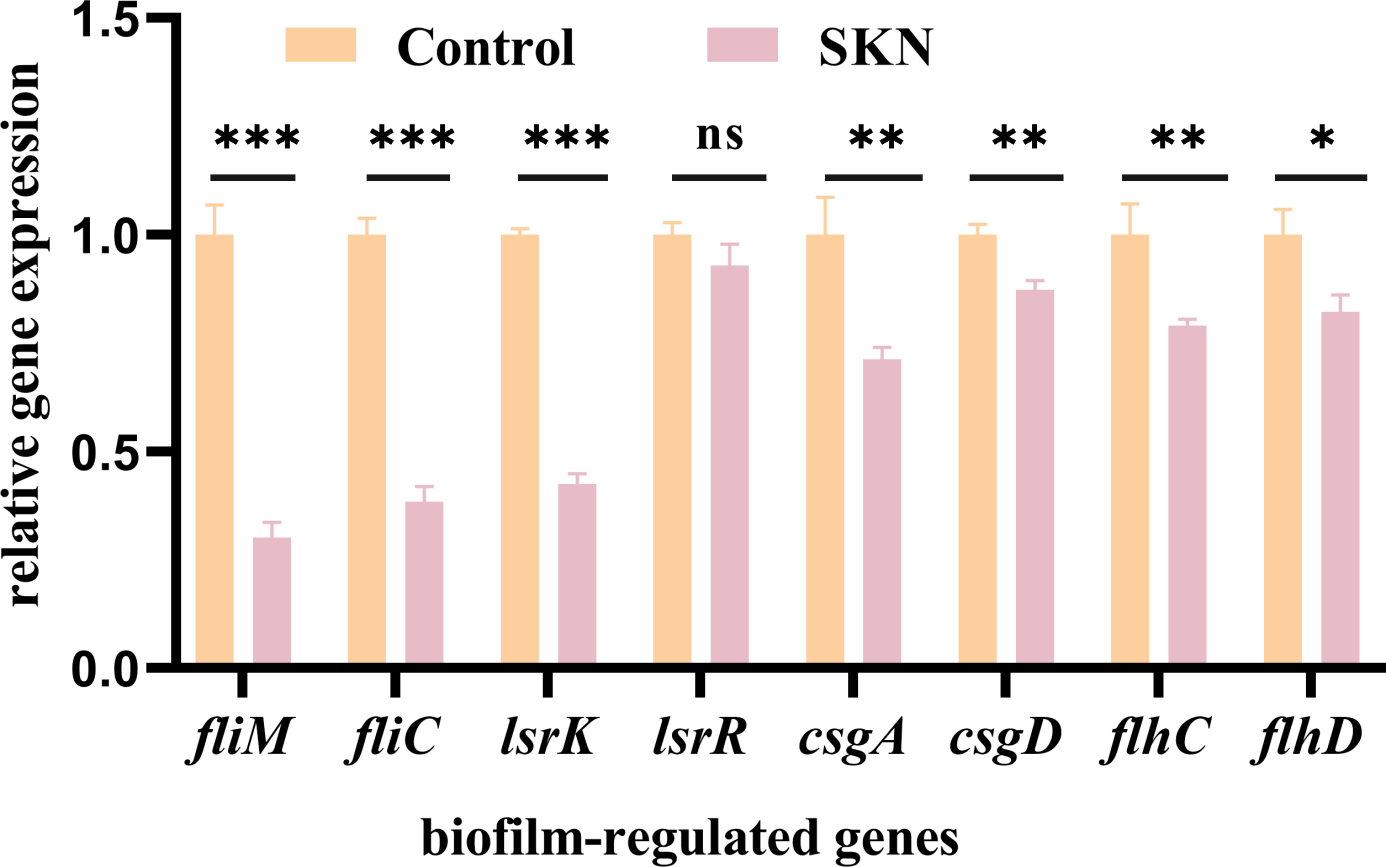
Effects of shikonin on transcription of biofilm-regulated genes. Significance was analyzed using a two-sample *t* test by comparing it with the control group. ***P < 0.001, **P  <  0.01, *P  <  0.05, ns, no significance; SKN, shikonin (32 µg/mL).

We next tested the effect of shikonin on the transcription of genes implicated in biofilm regulation in Col-R *E. coli* by qRT-PCR. As depicted in Fig. 3, shikonin (32 µg/mL) significantly inhibited the transcription of curli-related genes (*csgA* and *csgD*) (*P* < 0.01), flagella-formation genes (*flhC*, *flhD*, *fliC*, and *fliM*) (*P* < 0.01, *P* < 0.05, *P* < 0.001, and *P* < 0.001, respectively)*,* and QS-related genes (*lsrK* and *lsrR*) (*P* < 0.001 and ns).

### *In vivo* efficacy of colistin/shikonin in *G. mellonella*

**Fig 4:**
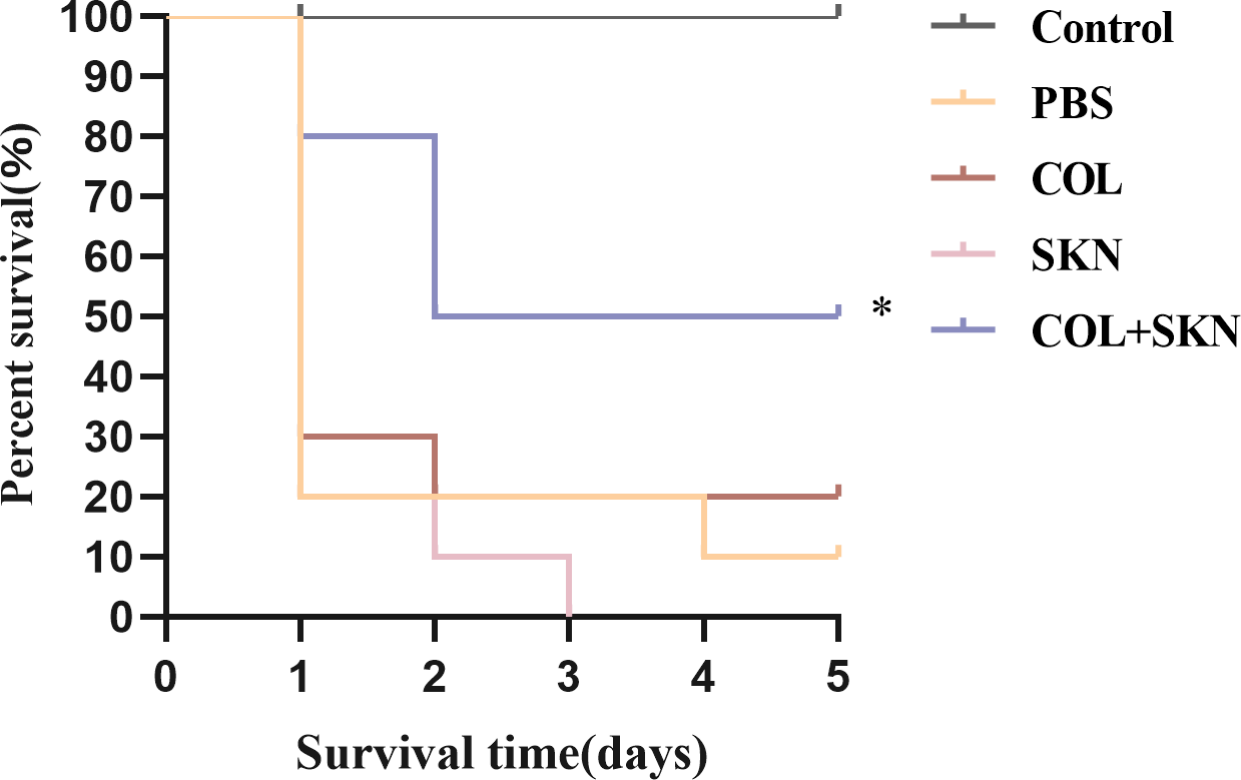
Effects of colistin or shikonin monotherapy or colistin/shikonin combination on the survival of *Galleria mellonella* infected with DC 4887. Significance was analyzed by log-rank test. *P  <  0.05, ns, no significance (all compared with control). COL, colistin; SKN, shikonin.

**Fig 5:**
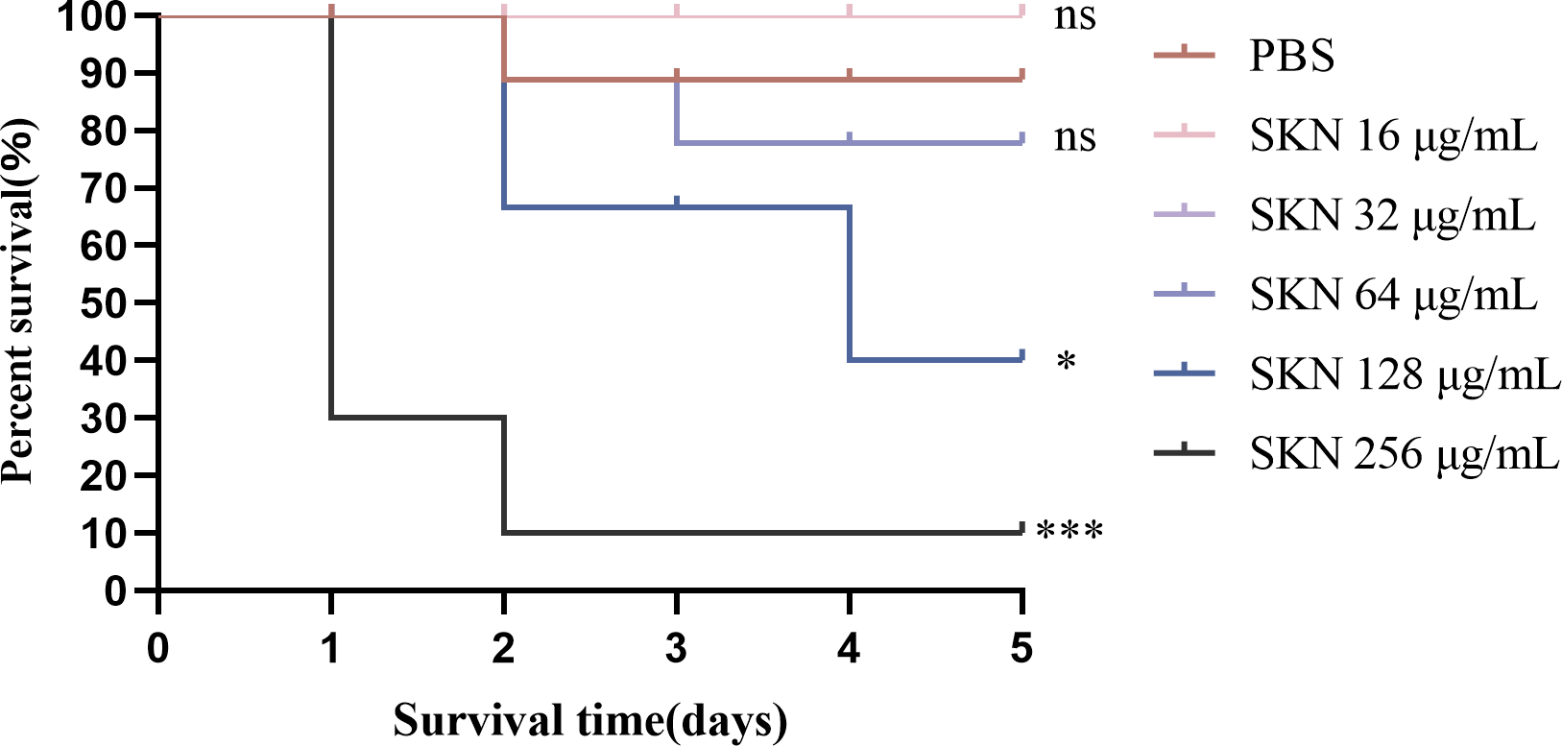
*In vivo* toxicity of different concentrations of shikonin on *Galleria mellonella*. Significance was analyzed by log-rank test. ***P < 0.001, *P  <  0.05, ns, no significance (all compared with control). SKN, shikonin (16–256 μg/mL).

As depicted in Fig. 4, larvae survival decreased to less than 20% within 5 days in the control group, colistin alone and shikonin alone group. In contrast, 50% survival was observed after 5 days of combination treatment of colistin and shikonin (*P* < 0.05). Fig. 5 shows the toxicity of shikonin, the survival rate of larvae injected with 256 or 128 µg/mL shikonin was significantly lower than 50% (*P* < 0.001 and *P* < 0.05, respectively). However, shikonin concentrations ≤64 µg/mL did not impact larval survival, shikonin did not affect larval survival rate, with no significant difference compared to the PBS-only group (ns).

### Membrane integrity change

**Fig 6:**
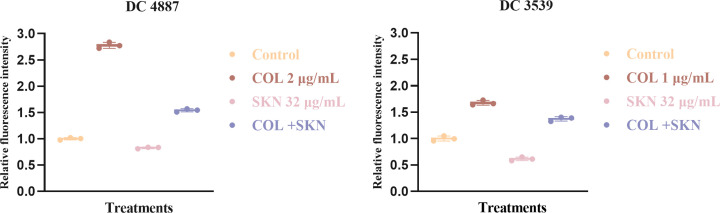
Fluorescence intensity of NPN uptake in DC 4887 and DC 3539. COL, colistin (1 or 2 µg/mL); SKN, shikonin (32 µg/mL).

**Fig 7:**
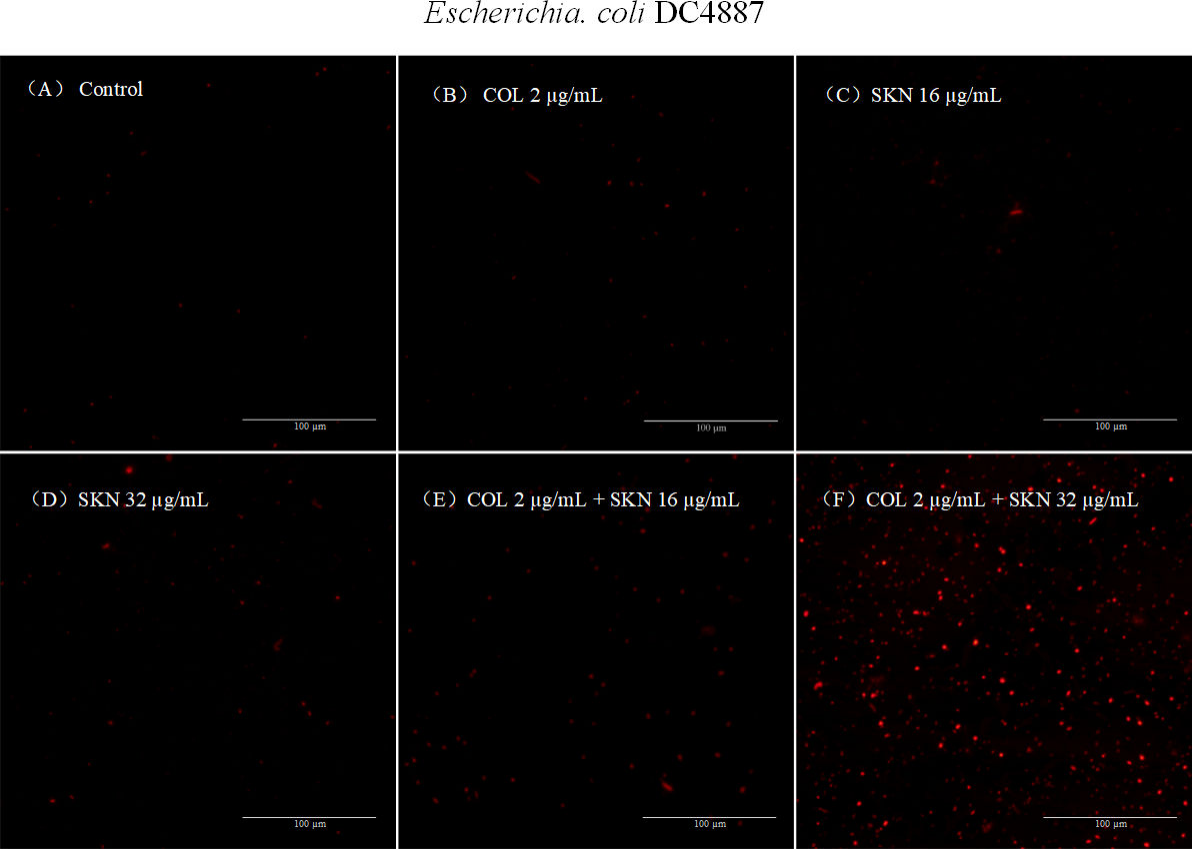
Fluorescence microscope images of PI membrane permeabilization in DC 4887. (**A**) Cells without any treatments with intact cell membranes showed no red fluorescence. (**B–D**) Cells treated with colistin or shikonin alone showed little fluorescence. (**E and F**) Cells treated with colistin/shikonin combination showed increased fluorescence with destroyed cell membranes. COL, colistin (2 µg/mL); SKN, shikonin (16 or 32 µg/mL).

NPN and PI were used to evaluate changes in outer and inner membrane integrity of bacterial cells, respectively. NPN is a hydrophobic fluorescent probe, when the OM is permeabilized by cationic compound (e.g., colistin), NPN is taken up by cells and becomes fluorescent in the hydrophobic interior of the membrane (24). In NPN uptake assays, exposure to colistin enhanced the fluorescence of NPN-probed cells (Fig. 6). In contrast, bacteria treated with 32 µg/mL shikonin alone exhibited only subtle changes in fluorescence intensity. PI is thought to stain only cells with damaged inner membranes (23). In PI assays, treatment with either colistin or shikonin resulted in little red fluorescence (Fig. 7B throuigh D). However, co-treatment with colistin and shikonin elicited a marked increase in red fluorescence (Fig. 7E and F).

### Intracellular ROS production

**Fig 8:**
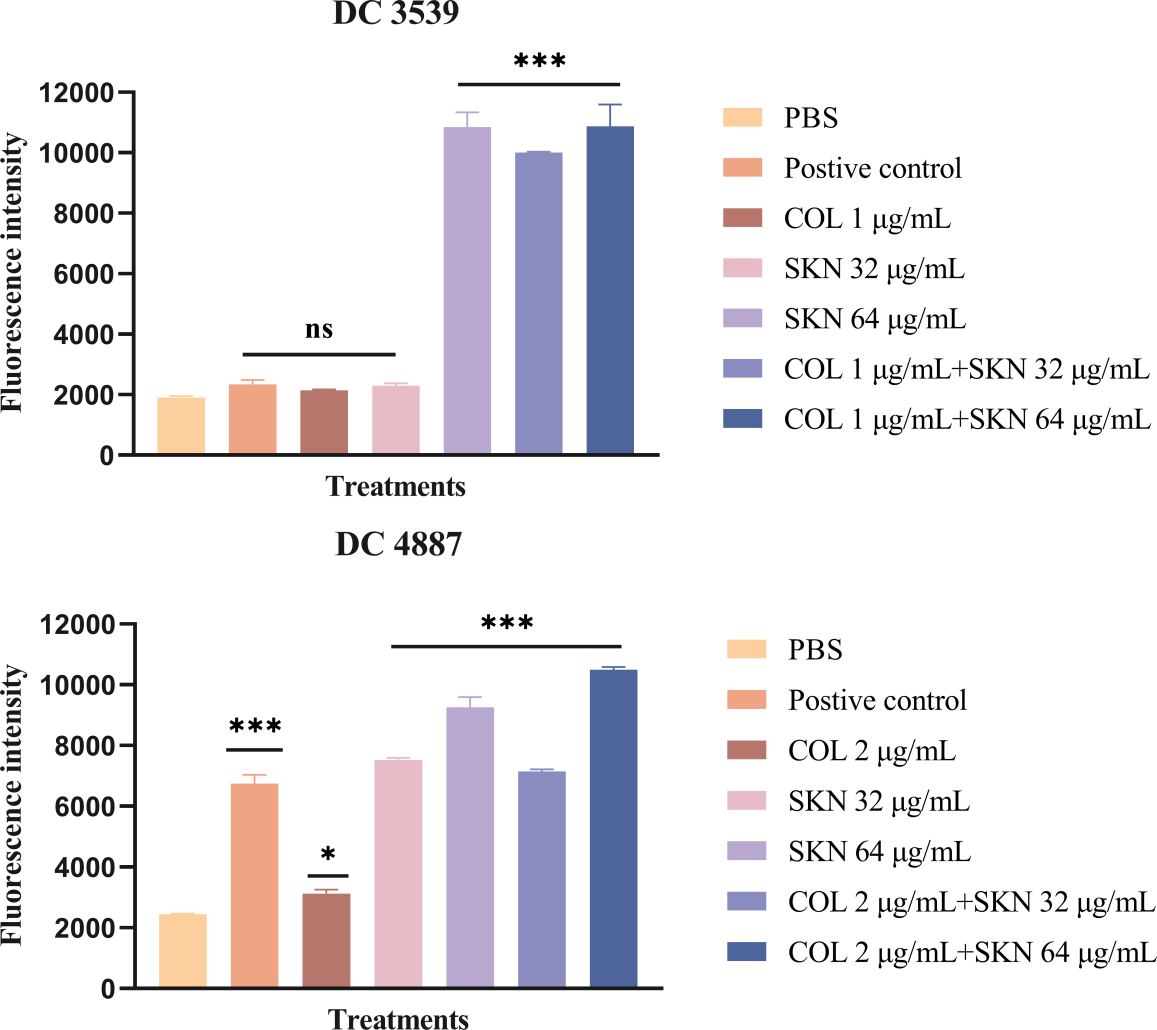
Reactive oxygen species (ROS) production in DC 4887 and DC 3539 after treatment. Significance was analyzed using one-way ANOVA by comparing it with the control group. ***P < 0.001, *P  <  0.05, ns, no significance. COL, colistin (1 or 2 µg/mL); SKN, shikonin (32 or 64 µg/mL).

To measure intracellular ROS levels in *E. coli* after being treated with shikonin, DCFH-DA staining was performed. As shown in Fig. 8, compared to the PBS control, shikonin alone or in combination with colistin elicited a significant increase in ROS levels (*P* < 0.001). However, 32 µg/mL shikonin did not induce a significant rise in ROS levels for DC 3539 (ns), which may be attributed to differences between the two strains. Therefore, the results showed that shikonin increased the production of ROS in *E. coli*, which may be one of the reasons for its increased antibacterial activity of colistin.

### Effects of shikonin on the transcription of *mcr-1* genes

**Fig 9:**
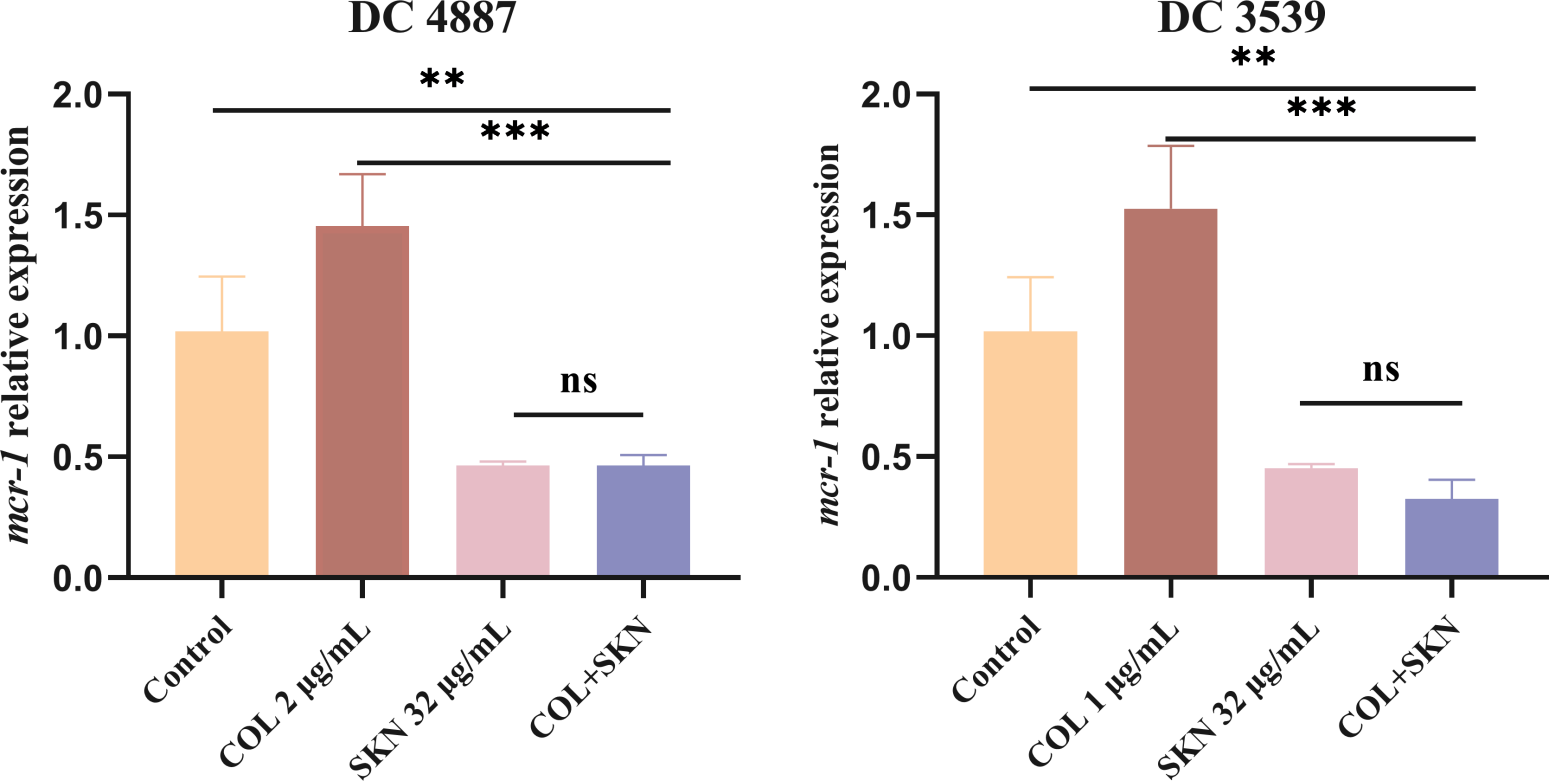
Relative expression of *mcr-1* genes of DC 4887 and DC 3539 after treatment. The data were expressed as mean  ±  S.D. **P  <  0.01, *P  <  0.05 (all compared with control). Significance was analyzed by one-way ANOVA. COL, colistin (1 or 2 µg/mL); SKN, shikonin (32 µg/mL).

Eight Col-R strains tested in this study all carried *mcr-1*. Therefore, qRT-PCR was used to analyze the effect of shikonin on the expression of *mcr-1* in DC 4887 and DC 3539. As shown in Fig. 9, the *mcr-1* expression level of bacteria treated with colistin only was upregulated compared to the control group (*P* < 0.05). However, in the treatment of shikonin alone or colistin/shikonin combination, gene expression was significantly downregulated (*P* < 0.05).

## DISCUSSION

The increasing prevalence of MDR GNB represents an urgent threat to public health globally. However, few novel antibiotics have been developed against MDR GNB, which exhibit resistance to most existing antimicrobial agents (29). Colistin is considered as the last-resort treatment option against MDR GNB, but colistin-resistant strains, especially colistin-resistant *E. coli*, are being identified with increasing frequency (8). Therefore, the development of new and efficacious antimicrobial therapies and/or innovative treatment strategies to combat infections caused by Col-R bacteria is of critical importance.

In this study, eight Col-R *E. coli* clinical isolates all exhibited MDR phenotypes and were resistant to most commonly used antibiotics, including cephalosporins, fluoroquinolones, carbapenems, aminoglycosides, nitrofurantoin, trimethoprim, and aztreonam. Colistin remains indispensable as a last line of defense, under such circumstances, strategies to potentiate its effects or surmount resistance are urgently warranted. The results from checkerboard method and time-kill curves demonstrated that shikonin can potentiate the antibacterial activity of colistin. The colistin/shikonin combination exhibited synergistic antibacterial effects against Col-R *E. coli in vitro.* More importantly, nephrotoxicity, neurotoxicity, and other side effects often occur during colistin treatment, thereby limiting the treatment range and dose of colistin (30). However, in our study, shikonin could significantly reduce the MIC value and dose of colistin required. Therefore, this indicates that combining colistin and shikonin may mitigate the side effects associated with the clinical use of colistin and broaden its therapeutic applications. Another problem with bacterial infections is the formation of biofilms, which poses a significant challenge to traditional antibiotics’ effectiveness (6). Therefore, we are trying to excavate an effective antibacterial strategy that exerts both antibacterial and antibiofilm effects. In this study, the colistin/shikonin combination could inhibit the formation of biofilms. Additionally, shikonin suppressed the transcription of curli-related genes (*csgA* and *csgD*), flagella-formation genes (*flhC*, *flhD*, *fliC*, and *fliM*), and QS-related genes. These findings suggest that shikonin may inhibit *E. coli* biofilm formation by downregulating the expression of curli, flagella, and QS-related genes.

To further evaluate the therapeutic efficacy *in vivo*, we established the *G. mellonella* infection model. The results demonstrate that the combination of two drugs also exerted synergistic effects *in vivo*. Previous studies reported that shikonin and its derivative imparted less toxicity towards treated tissues (13). Moreover, the concentration of shikonin used in this study did not impact the survival of *G. mellonella*. These findings suggest that the colistin/shikonin combination may have potential for *in vivo* applications. However, further meaningful work is required to illustrate the potential usefulness of this combination.

Subsequent to the aforementioned, we proceeded to explore the synergistic antibacterial mechanisms of the colistin/shikonin combination. In accordance with the findings of Lee et al. and Li et al., the antibacterial activity of shikonin against *S. aureus* has been shown to be affiliated with the affinity of the cell wall and the functional integrity of the bacterial membrane (15, 16). It was also demonstrated that shikonin can directly bind to peptidoglycan of the cell wall of MRSA and interfere with its integrity, whereas shikonin cannot bind to lipopolysaccharides (16). Accordingly, to gauge whether the synergistic antibacterial effect of colistin/shikonin in Col-R *E. coli* pertains to membrane permeability, we conducted the relevant experiments. An indispensable difference between Gram-negative bacteria (GNB) and Gram-positive bacteria (GPB) is that GNB possess an OM structure composed of lipopolysaccharide and glycerophospholipids, which plays a key role in resistance to antibiotic challenge by providing a low permeability barrier (31). Results from NPN and PI experiments indicated that shikonin alone had little effect on the OM of bacteria, whereas the effect of colistin/shikonin combination on the OM was principally dictated by colistin. Furthermore, shikonin or colistin alone had little effect on the inner membrane, whereas the colistin/shikonin combination could markedly increase the permeability of the inner membrane. ROS can have a damaging effect on the structure and functioning of proteins and may even cause bacterial cell death (32). Previous studies have shown that shikonin could target thioredoxin reductase of GNB, which plays an important role in maintaining redox homeostasis and regulating the production of ROS (33). Therefore, we investigated whether shikonin had an effect on ROS production in *E. coli*. The results indicated that shikonin could significantly augment the generation of ROS in *E. coli*.

Taken together, the augmented production of ROS and destruction of membrane integrity may constitute the principal reasons for the synergistic antibacterial effect of colistin and shikonin. Briefly, when shikonin acted on bacterial cells alone, its permeability across the OM was low, so shikonin could hardly play an antibacterial role. However, after the combination of shikonin and colistin, colistin interacted with the OM to increase the permeability, thereby increasing the entry of shikonin. Subsequently, shikonin interacted with the cell wall and inner membrane, resulting in membrane damage. At the same time, the induction of ROS by shikonin further led to cell membrane breakage and bacterial cell death. In this way, colistin and shikonin cooperate to play a synergistic antibacterial effect. Up to now, the main mechanism of colistin resistance in *E. coli* is plasmid-mediated mobile polymyxin resistance genes, among which *mcr-1* is the most common (34). In the study, shikonin inhibited the gene transcription levels of *mcr-1*, which may be one of the mechanisms by which shikonin enhances the antibacterial activity of colistin. The recent global spread of the *mcr-1* gene threatens the utility of colistin (35). In this context, this indicates that the combined strategy of colistin and shikonin has the prospect to alleviate *mcr-1*-mediated colistin resistance in *E. coli* and enhance the antibacterial effect of colistin.

### Conclusion

In summary, this study demonstrated the synergistic effect and antibacterial mechanisms of colistin in combination with shikonin against Col-R *E. coli*. Our study reveals that the colistin/shikonin combination may elicit synergistic antimicrobial effects by increasing cell membrane permeability, promoting intracellular ROS production, and inhibiting *mcr-1* gene expression. Our findings provide insights into the synergistic effects and antimicrobial mechanisms of the colistin/shikonin combination, and may further promote this strategy as an alternative therapy for infections caused by MDR *E. coli* or Col-R *E. coli*.

## SUPPLEMENTAL MATERIAL

Table S1 spectrum.01459-23-s0001.docx Supplemental table.

Table S2 spectrum.01459-23-s0002.docx Supplemental table.

ASM does not own the copyrights to Supplemental Material that may be linked to, or accessed through, an article. The authors have granted ASM a non-exclusive, world-wide license to publish the Supplemental Material files. Please contact the corresponding author directly for reuse.
