# Supplementary material for: An effective antimicrobial strategy of colistin combined with the Chinese herbal medicine shikonin against colistin-resistant Escherichia coli
Source: Microbiol Spectr. 2023 Oct 6;11(6):e01459-23. doi: 10.1128/spectrum.01459-23 (PMC10714725; doi:10.1128/spectrum.01459-23)
Supplement: Table S1 — Supplemental table. [file spectrum.01459-23-s0001.docx]

| Primer name | Sequence (5′-3′) | Product size(bp) | Reference |
| --- | --- | --- | --- |
| *mcr-1*-F | TGCTCCAAAATGCCCTACAGACC | 141 | (1) |
| *mcr-1*-R | TGCCCCAAGTCGGATAATCCAC |  |  |
| *16SrRNA*-F | TGTCGTCAGCTCGTGTTGTG | 130 |  |
| *16SrRNA*-R | ATCCCCACCTTCCTCCAGTT |  |  |
| *gapA-*F | CCAGGACATCGTTTCCAAC | 103 | (2) |
| *gapA-*F | GGTGGTCATCAGACCTTCG |  |  |
| *csgA-*F | CAGATGTTGGTCAGGGCTCAGATG | 127 |  |
| *csgA-*R | CCGCCACCGAATTGTTTAACTGTC |  |  |
| *csgD-*F | TGATGAACAACGAACGAGCGATCTC | 146 |  |
| *csgD-*R | GCTTGCCAGTTACCTGATTACACATTC |  |  |
| *flhC-*F | ATGCTGCCATTCTCAACCGACTG | 117 |  |
| *flhC-*R | CGCATCGACGCCATTACACAAAC |  |  |
| *flhD-*F | CGTTAGCGGCACTGACTCTTCC | 107 |  |
| *flhD-*R | TTGCGTCAACTGAGTAATCGTCTGG |  |  |
| *fliC-*F | TTACCAACCTGAACAACACCACTACC | 90 |  |
| *fliC-*R | ACATATTGGACACTTCGGTCGCATAG |  |  |
| *fliM-*F | CCGACCAACCTGAACCTTATCCATC | 88 |  |
| *fliM-*R | CCACGGCGATAAACACCAGACTC |  |  |
| *lsrK-*F | GATGAACCTACCGCCTCGCTTAC | 90 |  |
| *lsrK-*R | AACAATACCCACGCCAGTAGCAAG |  |  |
| *lsrR-*F | ACCACAACAGATGCTGGCGATTG | 143 |  |
| *lsrR-*R | GCTGCCCGATTCCCGTCATATAAG |  |  |

1. Wang YM, Kong LC, Liu J, Ma HX. 2018. Synergistic effect of eugenol with Colistin against clinical isolated Colistin-resistant Escherichia coli strains. Antimicrob Resist Infect Control 7:17.

2. Bai Y, Wang W, Shi M, Wei X, Zhou X, Li B, Zhang J. 2022. Novel Antibiofilm Inhibitor Ginkgetin as an Antibacterial Synergist against Escherichia coli. Int J Mol Sci 23.

**Table S1 Primers used in the study**
