# Supplementary material for: An effective antimicrobial strategy of colistin combined with the Chinese herbal medicine shikonin against colistin-resistant Escherichia coli
Source: Microbiol Spectr. 2023 Oct 6;11(6):e01459-23. doi: 10.1128/spectrum.01459-23 (PMC10714725; doi:10.1128/spectrum.01459-23)
Supplement: Table S2 — Supplemental table. [file spectrum.01459-23-s0002.docx]

| Isolates | Isolation Date | Patient age | Patient Gender | Sample | Ward |
| --- | --- | --- | --- | --- | --- |
| DC3539 | 26/03/2015 | 82 | M | Drainage fluid | Gastrointestinal Surgery |
| DC3599 | 13/04/2015 | 72 | M | Sputum | Respiratory Internal Medicine |
| DC3737 | 05/05/2015 | 52 | M | Wound | Orthopedics |
| DC3846 | 28/05/2015 | 77 | F | Urine | Urine Urology |
| DC4887 | 23/02/2016 | 63 | M | Urine | Urine Urology |
| DC5286 | 23/05/2016 | 82 | F | Urine | Endocrinology |
| DC7333 | 18/05/2016 | 32 | M | Drainage fluid | ICU |
| DC8277 | 08/01/2018 | 65 | F | Urine | Hematology |

**Table S2 Clinical background of eight Col-R *E. coli* isolates**

Abbreviations: M, male; F, female.
